# The Role of Estrogen and Thyroid Hormones in Zebrafish Visual System Function

**DOI:** 10.3389/fphar.2022.837687

**Published:** 2022-02-28

**Authors:** Annastelle Cohen, Jeremy Popowitz, Mikayla Delbridge-Perry, Cassie J. Rowe, Victoria P. Connaughton

**Affiliations:** ^1^ Department of Biology, American University, Washington, DC, WA, United States; ^2^ Department of Chemistry, American University, Washington, DC, WA, United States; ^3^ Center for Neuroscience and Behavior, American University, Washington, DC, WA, United States

**Keywords:** *Danio rerio* (zebrafish), retina, estradiol, T3, T4, development

## Abstract

Visual system development is a highly complex process involving coordination of environmental cues, cell pathways, and integration of functional circuits. Consequently, a change to any step, due to a mutation or chemical exposure, can lead to deleterious consequences. One class of chemicals known to have both overt and subtle effects on the visual system is endocrine disrupting compounds (EDCs). EDCs are environmental contaminants which alter hormonal signaling by either preventing compound synthesis or binding to postsynaptic receptors. Interestingly, recent work has identified neuronal and sensory systems, particularly vision, as targets for EDCs. In particular, estrogenic and thyroidogenic signaling have been identified as critical modulators of proper visual system development and function. Here, we summarize and review this work, from our lab and others, focusing on behavioral, physiological, and molecular data collected in zebrafish. We also discuss different exposure regimes used, including long-lasting effects of developmental exposure. Overall, zebrafish are a model of choice to examine the impact of EDCs and other compounds targeting estrogen and thyroid signaling and the consequences of exposure in visual system development and function.

## Introduction

The impacts of endocrine manipulation often result in a cascade of effects at the biomolecular level, reaching outside a single pathway, and many non-endocrine organs, such as kidney and gut, secrete hormones. Further, neuronal development, and sensory system development, are dependent on hormones. Recent work has revealed the importance of thyroid hormones (THs), estrogen, and their receptors in visual system development. Both hormones are able to cross cell membranes, bind intracellular receptors, and affect subsequent pathways and/or gene expression. Disruption of either estrogenic or thyroidogenic pathways, by clinical treatments or environmental endocrine disrupting compounds (EDCs), impact the visual system directly or indirectly, and early developmental exposure to endocrine disruptors can have long-term deleterious effects. In addition to the epidemiological significance of endocrine disruption in humans, these effects also impose consequences on ecological systems at a population level as visual perception is essential for the success, survival, and reproduction of many organisms. The purpose of this review is to compare/contrast the role(s) of thyroid hormone and estrogen in the proper function and development of the visual system in the zebrafish animal model.

### Zebrafish

Zebrafish, *Danio rerio*, a small freshwater tropical fish native to Southeast Asia, are an existing vertebrate model for a variety of disciplines, including endocrinology, toxicology, developmental biology, and vision. Adult zebrafish measure 2.5–4 cm in length and, due to their small size, can be housed in large numbers at a low-cost relative to other available model organisms. The zebrafish genome has been sequenced in its entirety (https://www.sanger.ac.uk/data/zebrafish-genome-project/), making this species valuable for investigation of various disorders and disease. Zebrafish have more than 26,000 protein-coding genes and 70% of human genes have at least one obvious zebrafish orthologue (Howe et al., 2013). Mutant strains and transgenic lines can be easily and quickly produced and assessed using large-scale genetic screens. Further, large clutch sizes of externally developing, transparent embryos are amenable to exposure studies as compounds are administered directly into tank water resulting in behavioral and/or physiological responses that can be recorded.

In addition to the technical and practical advantages of zebrafish, they serve as a powerful model organism for studying visual system development, function, and underlying mechanisms of disease. Zebrafish eyes are similar in anatomy, circuitry, physiology, and gene expression to humans (Bibliowicz et al., 2012). The zebrafish retina contains similar cell types and circuitry to the human retina, and retina-specific diseases observed in humans, such as red color blindness (Brockerhoff et al., 1997) and congenital stationary night blindness (Peachey et al., 2012) occur in and are modeled with zebrafish.

Zebrafish have also been used to study early life and adult effects of hormones, at both organizational and activational levels. Zebrafish nervous and endocrine systems (Tata, 2005) are also similar to humans from development throughout adulthood (Kimmel et al., 1995; Kishida and Callard, 2001; Gerlai, 2016). Studies with EDCs reveal effects on development, reproduction, sensory systems, cell proliferation, and heart formation. EDC exposure has been linked to obesity (Hatch et al., 2010; Heindel et al., 2015), metabolic and reproductive issues (Wada et al., 2007; Casals-Casas and Desvergne, 2011), and neurological disorders (Kajta and Wojtowicz, 2013). Specific to this review, EDCs can affect the brain/neurogenesis (Kishida et al., 2001; Hamad et al., 2007; Pelligrini et al., 2007; Diotel et al., 2013), including negatively impacting the visual system (Dong et al., 2006; Hamad et al., 2007; Wang et al., 2012).

Here, we focus on the specific effects of estrogen and thyroid hormone signaling in retinal development and function. We begin with a general description of eye development and structure in zebrafish and signaling by estrogen and thyroid hormones. We then discuss the role of estrogenic and thyroidogenic signaling in vision. We conclude with a summary of these effects, revealing significant crosstalk among these two systems that is important for proper visual function.

### Visual System Development

**FIGURE 1:**
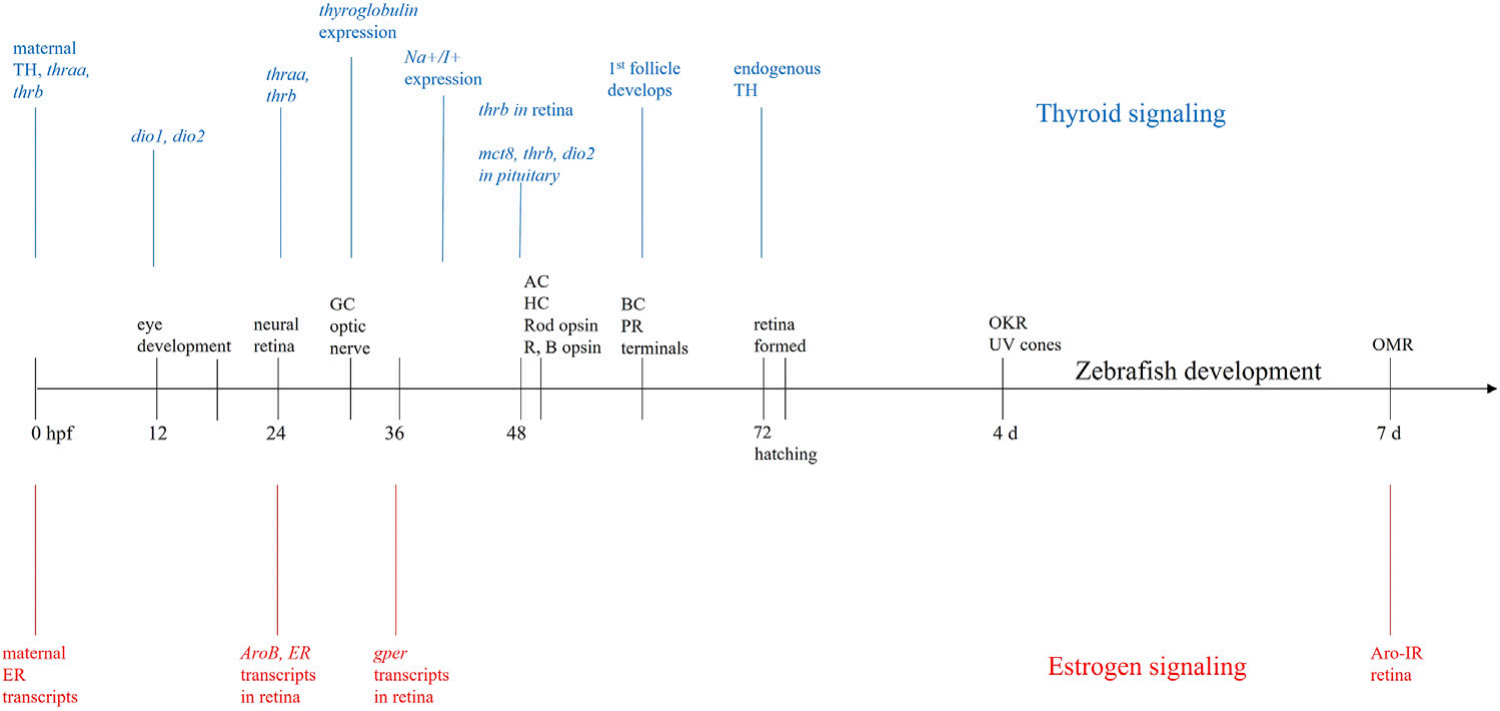
Developmental timeline. Sequence of events in zebrafish eye and retinal development (black) beginning at fertilization (0 h postfertilization—hpf) and continuing until 7 days (days) postfertilization. During this time, maternally derived transcripts for estrogen receptors (ER—red) and maternally derived thyroid hormones (TH—blue) are present in yolk. At 24 hpf, when neural retina is developing, expression of ER and aromatase (*AroB*) are present in retina, and expression of the genes for both thyroid receptors (*thraa, thrb*) begins. Expression of G-protein coupled ER (*gper*) begins ∼36 hpf; at 48 hpf *thrb* is expressed in retina. At hatching (72 hpf), there is endogenous production of both TH and estrogen. The overlap and concurrent development of thyroid, estrogen, and retinal development, suggests these hormones are important for proper retinal/visual system development. GC = ganglion cell, AC = amacrine cell, HC = horizontal cell, R = red cone, B = blue cone, BC = bipolar cell, PR = photoreceptor, OKR = optokinetic response, OMR = optomotor response.

Morphogenesis of the zebrafish eye occurs very rapidly between 12 and 24 h post fertilization (hpf) and the structure of the eyes is thought to be fully realized by 36 hpf (Schmitt and Dowling, 1994; Schmitt and Dowling, 1999). Within the retina, differentiation first occurs in a ventronasal patch near the optic nerve, and like most other vertebrates, moves from inner to outer retina (Schmitt and Dowling, 1999). At 32 hpf, ganglion cells begin to form and the optic nerve exits the retina. By 50 hpf, amacrine and horizontal cells in the inner nuclear layer begin to differentiate. Bipolar cells in the inner nuclear layer begin to differentiate at 60 hpf, as do rod and cone synaptic terminals. At 74 hpf, the zebrafish eye is fully developed (Schmitt and Dowling, 1999). Optokinetic responses can be recorded from zebrafish larvae as young as 4 days postfertilization (dpf) (Neuhauss, 2003; Brockerhoff, 2006), and vision-based optomotor responses are reliably recorded at 7 dpf (Clark, 1981; Bilotta et al., 2002; Bahadori et al., 2003; Orger et al., 2004; Muto et al., 2005) (Figure 1).

The adult zebrafish retina includes four cone types (R—red, G—green, B—blue, UV—ultraviolet) arranged in an orderly mosaic. These cone types are present in larval retinas, though the mosaic is less organized (Allison et al., 2010). During embryogenesis, photoreceptors develop at 43–48 hpf and opsins in rods, R and B cones are first detected at ∼50–52 hpf (Raymond et al., 1995; Tsujikawa and Malicki, 2004). The presence of four cone types confers rich color processing abilities (Meier et al., 2018), in both larvae and adults, with a diversity of color-evoked responses seen in second-order horizontal cells (Connaughton and Nelson, 2010) as well as third-order amacrine (Torvund et al., 2016) and ganglion cells (Connaughton and Nelson, 2010). Color responses in zebrafish retinal bipolar cells have not been recorded; though anatomical analysis of dendritic connections with cone pedicles suggests multiple spectral types (Li et al., 2012). Signal transduction in the retina is highly conserved across vertebrates, with the connections of retinal neurons, overall layered organization, parallel ON- and OFF-pathways, and excitatory glutamatergic inputs within the vertical transduction pathway (photoreceptors to bipolar cells to ganglion cells) observed in both zebrafish and humans. GABAergic, glycinergic, and dopaminergic cells are also present. A difference between zebrafish and mammals is seen in brain circuitry: the optic tectum, a midbrain structure, is responsible for all higher order visual processing in zebrafish. In humans, the midbrain LGN (lateral geniculate nucleus) receives and processes retinal inputs before projecting to visual cortex (V1) (Haynes et al., 2005). Though lacking V1, the zebrafish optic tectum is well developed and capable of cortical-level processing of visual stimuli, such as stimulus detection and orientation (Hunter et al., 2013), escape behaviors (Dunn et al., 2016), and prey capture (Muto and Kawakami, 2013). Thus, the overall similarities in anatomy and circuitry between zebrafish and humans, coupled with genetic techniques that can be easily applied, allows zebrafish to serve as a convenient and relevant model for testing the effects of endocrine disrupting compounds (EDCs) and for assessing deficits in visual physiology and behavior (Link and Collery, 2015).

### Estrogen Localization, Signaling, Receptor Types

Classically, estrogen production occurs in the gonads and, to a lesser extent, the adrenal cortex, with release stimulated by the hypothalamic-pituitary axis. Estradiol (17β-estradiol or E2), the biologically relevant estrogen, is synthesized directly from the aromatization of testosterone by the enzyme aromatase (estrogen synthase), a product of the *cyp19* gene. E2 is released from these glands directly into the bloodstream and, as it is best known for its roles in reproductive functions, is often referred to as a gonadal sex steroid (Menuet et al., 2005; Mccarthy, 2008). However, we have since learned that E2 synthesis and action are not restricted to reproductive tissues: E2 is locally produced via aromatase in a variety of non-reproductive tissues and throughout the nervous system (Lephart, 1996), inducing potent pleiotropic effects on central nervous system development, maturation, and function (Menuet et al., 2005).

For example, E2’s effects extend to the visual system. The retina expresses aromatase, and estrogen receptors (ERs) are found in all retinal layers across vertebrate species (Gellinas and Callard, 1993; Callard et al., 2001; Cascio et al., 2007; Cascio et al., 2015), pointing to a conserved functional significance of local E2 synthesis and action. Indeed, E2 is neuroprotective in retina, preventing excitotoxic cell death and protecting against retinal degeneration in humans (Cascio et al., 2007). E2 also influences eye structure and function and the incidence of many ocular diseases (Cascio et al., 2007) and changes in E2 levels from aging or hormone therapies are associated with neurodegenerative retinal diseases and visual complications (Cascio et al., 2015).

Developmentally, ER transcripts in newly fertilized zebrafish embryos are maternally derived (Bardet et al., 2002; Lassiter et al., 2002; Tingaud-Sequeira et al., 2004) with endogenous transcription beginning around 24–48 hpf (Figure 1) (Bardet et al., 2002; Lassiter et al., 2002; Tingaud-Sequeira et al., 2004) and corresponding with the onset of aromatase mRNA expression (Mouriec et al., 2009). By 7 dpf, aromatase can be detected in retina using immunocytochemistry (Le Page et al., 2011), suggesting local E2 expression.

#### Aromatase in the Fish Brain

Teleost fish have remarkably high levels of neural aromatase, a finding pioneered in longhorn sculpin (Callard et al., 1978) and goldfish (Pasmanik and Callard, 1988; Gellinas and Callard, 1993; Callard et al., 1995) and confirmed in other teleosts (Trimmers et al., 1987; Borg et al., 1989; Mayer et al., 1991; Gonzalez and Piferrer, 2002), including zebrafish (Kishida and Callard, 2001; Sawyer et al., 2006). It is estimated that teleost neural aromatase is about 100 to 1000-fold greater when compared to mammals and birds (Pellegrini et al., 2005), and this high aromatase is thought to be involved in the regenerative abilities and plasticity of the teleost brain, optic nerve, and eye (Hamad et al., 2007). Furthermore, estrogens and estrogenic compounds upregulate the expression of developmental aromatase (Menuet et al., 2005; Sawyer et al., 2006), allowing neural aromatase in teleosts to serve as an indicator of estrogen signaling and modulation (Kishida et al., 2001).

Another unique feature of teleosts is that neural aromatase is exclusively expressed in a single and distinct cell type: radial glial cells (Menuet et al., 2005). These radial glial cells serve as progenitor cells that are essential in neurogenesis, where in mammals they act as embryonic neural stem cells that disappear shortly after birth (Schmidt and Scholpp, 2013). While these cell types are similarly important during zebrafish neurogenesis, they also persist into adulthood, continuing to express high levels of aromatase, proliferate, self-renew, and generate new neurons (Schmidt and Scholpp, 2013). Therefore, adult zebrafish seem to possess embryonic mammalian features in terms of neurogenesis, allowing them to serve as sensitive models for estrogen signaling and the effects of disruption (Le Page et al., 2011).

Zebrafish have two separate and distinct aromatase expressing genes that have subdivided expression domains. The *cyp19a* gene encodes aromatase A (*AroA*) which is primarily expressed in the gonads, whereas the *cyp19b* gene encodes aromatase B (*AroB*) which is expressed in neural tissues, including the brain and retina (Callard et al., 2001; Menuet et al., 2005). In the retina of goldfish, *AroB* has been detected in horizontal, bipolar, and amacrine cells, and within ganglion cell projections to the brain from the optic nerve and tract (Callard et al., 1995). In support of a functional role of aromatase in the visual system, developmental exposure to known aromatase inhibitors causes thinning of retinal layers, delayed eye growth, and deficits in visually-guided behaviors (Hamad et al., 2007; Gould et al., 2017). Taken together, the localization of *AroB* in the retina and the anatomical and visual impacts from aromatase inhibition suggest a key role for E2 in the development and function of the visual system.

#### Estrogen Receptors and Signaling

Sequencing and phylogenetic analyses of human and zebrafish ERs reveal conserved functional motifs, high sequence homology, particularly in the DNA binding domain (C domain) (Bardet et al., 2002; Menuet et al., 2002) and near identical exon numbers and lengths (Lassiter et al., 2002). Further, human and teleost ERs exhibit similar binding characteristics (Thomas et al., 2010) and share the same intracellular signaling cascades (Thomas et al., 2010) and mechanisms of transcriptional activation (Klinge, 2001; Bardet et al., 2002). Thus, while we acknowledge that there may be species-specific differences in timing of events, the general signaling pathways and mechanisms of estrogen signaling are highly conserved across vertebrates (Klinge, 2001).

The actions of E2 are primarily mediated through two intracellular ERs, ERα and ERβ, that act as ligand-activated transcription factors to modulate estrogen target gene activity (Menuet et al., 2002). In the classical signaling pathway, intracellular ERs will form homo- or heterodimers upon E2 binding and translocate to the nucleus (Cascio et al., 2015). Once there, dimerized receptors bind to estrogen response elements (EREs) in promoter regions of DNA and recruit specific cofactors to alter gene expression (Belcher and Zsarnovszky, 2001; Mccarthy, 2008). E2 targets genes expressed in the retinal photoreceptor layer including *grk7a* and *pde6ga* (Hao et al., 2013). *grk7a*, or G-protein-coupled receptor kinase 7a, is involved in visual perception and phototransduction; *pde6ga* is predicted to be involved the activation of MAPK activity (Vogalis et al., 2011; Thisse and Thisse, 2014). E2 increases the expression of these genes, thus, lower E2 levels would decrease expression, causing lowered photosensitivity or lower level of function overall in retina (Vogalis et al., 2011; Thisse and Thisse, 2014).

In addition to direct interaction with EREs, E2-activated nuclear ERs can also regulate transcription via an indirect genomic mechanism by associating with and influencing activity of transcription factors, including stimulating protein 1 (SP-1), activator protein 1 (AP-1), nuclear factor-κB (NF-κB), and c-jun (Cui et al., 2013). Estrogen receptors can also participate in indirect “extranuclear signaling” through membrane-localized ERs in association with cytosolic kinases and growth factor signaling components to mediate rapid estrogenic effects (Levin, 2002). This extranuclear pathway can initiate multiple cytoplasmic signaling cascades that involve the downstream activation of MAPK/ERK, PI3K/AKT, and cAMP/PKA, which can ultimately also lead to transcriptional changes (Cortez et al., 2013).

The ERα and ERβ isoforms have distinct functions (Mccarthy, 2009), developmental expression patterns, tissue distributions, genes, and affinities for E2 (Menuet et al., 2002). However, the presence of ERα and ERβ in retina has been observed in many vertebrate animals, including rats, bovines, humans, and teleosts (Kobayashi et al., 1998; Cascio et al., 2015). In zebrafish, there are two forms of ERβ (zfERβ1 and zfERβ2), which likely resulted from a duplication event in the teleost lineage (Menuet et al., 2002). In development, it is thought that ERβ1 is most highly expressed (Paige et al., 1999; Froehlicher et al., 2009). All three ERs (zfERα, zfERβ1, and zfERβ2) are detected in zebrafish eyes where they begin to be highly expressed 24-48hpf (Menuet et al., 2002; Tingaud-Sequeira et al., 2004; Mouriec et al., 2009) (Figure 1).

E2 can also bind to a membrane-bound G protein-coupled estrogen receptor (GPER) to elicit indirect rapid non-genomic signaling (Belcher and Zsarnovszky, 2001). GPER binding E2 activates cAMP through adenylyl cyclase, which causes downstream activation of MAPK and CREB pathways, promoting neuronal growth and survival (Shi et al., 2013; Roque and Baltazar, 2019). GPER activation also promotes the activity of kinases involved in neuronal protection, such as PI3K/AKT (Roque and Baltazar, 2019).

Although the nervous system effects of E2 are largely attributed to classical ER genomic signaling, increasing evidence suggests that E2 mediated GPER activation is also involved (Luo and Liu, 2020). During zebrafish embryogenesis, GPER mRNA and protein shows a wide distribution throughout the central nervous system and can be detected from fertilization to 72 hpf, with high levels of expression occurring after 24 hpf (Jayasinghe and Volz, 2011; Shi et al., 2013). *gper* has also been detected in the zebrafish eye at 36 hpf and more recently in the retina, optic tract, and in nuclei of primary and secondary visual pathways of adult goldfish (Mangiamele et al., 2017). Further, genes involved in the MAPK/ERK pathway are present in zebrafish retina at various stages of development (Krens et al., 2006). GPER also plays a functional role as knockdowns induce apoptosis, decrease proliferation of brain cells, and cause abnormal development of sensory neurons (Shi et al., 2013). The presence of *gper* and downstream genes in the retina of embryonic zebrafish and the functional deficits of GPER knockdown suggest that E2 might rapidly modulate sensory processes via this non-genomic signaling pathway. Therefore, it appears that E2 exerts its effects in neurogenesis and neuroprotection using both long-term, transcriptional, and rapid, non-genomic mechanisms.

### Thyroid Hormone Localization, Signaling, Receptor Types

The adult thyroid gland releases two thyroid hormones (THs): tri-iodothyronine (T3) and tetra-iodothyronine, or thyroxine (T4). Both T3 and T4 require iodine, which is taken up from the blood stream and, after binding tyrosine, is bound to thyroglobulin for storage within thyroid follicles (Sellitti and Suzuki, 2014). Though a greater amount of T4 is released, it is converted to T3 after release and T3, with greater affinity for thyroid receptors, is the more active form. Conversion of T4 and/or T3 occurs through the activity of three deiodinase enzymes: deiodinase type 2 (Dio2 or D2) converts T4 to T3, deiodinase type 3 (Dio3 or D3) inactivates T3 by converting it to reverse T3 (rT3) (Darras et al., 1999), and Dio1 (D1) performs both reactions, though it is considered the least efficient of the three (Bianco and Kim, 2006; Darras et al., 2011). All three deiodinases are present in zebrafish, and Dio2 is the major isoform producing useable T3 (Porazzi et al., 2009). Thyroid hormones impact all cells in the body, as they are important for growth and metabolic rate, and are involved in a variety of pathways during development (Silva et al., 2017).

In particular, THs are required for proper brain/CNS development (Boas et al., 2006; Darras et al., 1999; Howdeshell, 2002). In humans, initial TH levels are of maternal origin, with endogenous production occurring after 10–12 weeks gestation (Darras et al., 1999; Kohrle and Fradrich, 2021; Howdeshell, 2002). D3 activity in the placenta and fetus maintains constant fetal T3 levels (Patrick, 2009). Zebrafish embryos also have measurable levels of TH of maternal origin (Figure 1) (Silva et al., 2017; Gothie et al., 2019), resulting in stable whole body T3 and T4 levels until 60–72 hpf (Chang et al., 2012). At ∼72 hpf, endogenous TH synthesis begins (Porazzi et al., 2009; Darras et al., 2011; Gothie et al., 2019; Vancamp et al., 2019) causing internal TH levels to increase significantly, peaking at 10 dpf (T3) and 21 dpf (T4) (Chang et al., 2012). A more recent study measuring T3 and T4 levels using fluorescent antibodies found hormone levels peak earlier, at 6 dpf, and then decrease (Rehberger et al., 2018). Prior to hatching, thyroglobulin expression begins at 32 hpf and Na^+^/I^+^ symporter expression starts at 40 hpf (Alt et al., 2006). The first thyroid follicle is evident ∼55–60 hpf (Alt et al., 2006) and follicles can be clearly seen at 72 hpf, which coincides with the onset of endogenous T4 production (Porazzi et al., 2009). Interestingly, this development and early functioning of the thyroid gland does not require thyroid-stimulating hormone (TSH) (Alt et al., 2006) and is, therefore, independent of the hypothalamic-pituitary axis (Vancamp et al., 2019).

Altering T3 levels by knockdown of deiodinases disrupts eye development by decreasing eye size, reducing cone numbers, and altering visually guided responses in zebrafish (Houbrechts et al., 2016). Sensitivity of the retina to TH levels remains throughout life. Indeed, external application of T3 from 2 to 4 dpf alters cone opsin expression in exposed larvae, an effect also observed in juveniles exposed from 26 to 31 dpf (Mackin et al., 2019).

#### Thyroid Hormone Receptors and Signaling

T3 converted from T4 is transported into target cells via a high affinity membrane transporter (such as monocarboxylate transporter 8 or mct8) (Arjona et al., 2011), where it binds to a thyroid hormone receptor (TR). Similar to ERs and other members of the nuclear receptor superfamily, TRs act as ligand-activated transcription factors that influence transcription of target genes (Flamant et al., 2017). TRs bound to T3 form a dimer, commonly a heterodimer (Flamant et al., 2017), with the retinoid X receptor (TR/RXR) (Li et al., 2002; Li et al., 2004) or the retinoic acid receptor (TR/RAR) (Lee and Privalsky, 2005) before binding to a thyroid response element (TRE) on DNA to alter target gene transcription (Lazar et al., 1991). An interesting aspect of thyroid hormone signaling is that both RXR and RAR can also bind their natural ligand, retinoic acid, when bound to TR (Li et al., 2002; Li et al., 2004; Bohnsack and Kahana, 2013) and interactions between thyroid and retinoic acid signaling have been reported (Essner et al., 1997). In addition to this canonical genomic pathway, T3 can interact with plasma membrane integrin αvβ3 to initiate rapid intracellular signaling cascades involved in neuroprotection, growth, and apoptotic regulation, including MAPK (ERK1/2) and PI3K/AKT (Flamant et al., 2017).

In mammals there are 2 TR genes: TRα and TRβ (Bernal et al., 2003; Bernal, 2005). Zebrafish also have TRα and TRβ that respond to TH (Porazzi et al., 2009). In mammals, the TH receptor genes code for various protein products with TRα1, TRβ1, TRβ2, and TRβ3 able to bind to both T3 and to DNA (Bernal et al., 2003; Bernal, 2005). In zebrafish, one gene encodes TRβ (*thrb*), but two genes encode TRα (*thraa* and *thrab*) (Liu et al., 2000; Porazzi et al., 2009; Darras et al., 2011; Marelli et al., 2016), with all receptor isoforms expressed in retina. The *thraa* gene forms two proteins: TRαA-1 and TRαA1-2 (Porazzi et al., 2009; Darras et al., 2011), with TRαA-1 corresponding to mammalian TRα1 (Darras et al., 2011). *Thrb* encodes three isoforms: zTRβ1s (short), zTRβ1L (long) and zTRβ2 (Vancamp et al., 2019). All receptors bind T3 and are intracellular (Marelli et al., 2016).

During early embryogenesis, both *thraa* and *thrb* are expressed in zebrafish embryos (Liu et al., 2000; Porazzi et al., 2009; Vancamp et al., 2019), peaking at 18 hpf, then decreasing to undetectable levels until 24 hpf when expression again increases (Marelli et al., 2016). At 48 hpf, *thrb* is detectable in retina and it is still expressed in the eye and muscles of adult zebrafish (Marelli et al., 2016). Expression of *mct8* (Vancamp et al., 2019) and *Dio2* are also found in developing retina (Bohnsack and Kahana, 2013). Thus, though endogenous TH production does not begin until hatching, gene expression and/or development of thyroid signaling components are present much earlier, indicating high embryonic TH levels may drive expression of pathway components (Liu et al., 2000).

### Role of Estrogen in Visual Function

As noted above, development of the retina/visual system and estrogenic signaling occur simultaneously (Figure 1), suggesting an interaction between these two processes. Indeed, many animal studies have suggested that proper estrogenic signaling is critical for neurogenesis of the visual system: developmental manipulation of estradiol signaling or synthesis causes abnormal eye growth (Hamad et al., 2007; Hano et al., 2007), deficits in visually guided behaviors (Lovato et al., 2017; Crowley-Perry et al., 2021), and thinning and apoptosis in the retina (Dong et al., 2006; Hamad et al., 2007). Much of this research uses EDCs to determine the role of E2 in the visual system. Here, we discuss the visual system effects of EDCs acting as E2 agonists—BPA and EE2—and E2 antagonists—TBT and 4-OH-A.

Bisphenol-A (BPA) is a familiar, ubiquitous chemical (Arase et al., 2011) used primarily in the manufacture of polycarbonate and epoxy resins (Chapin et al., 2008) and it is present in plastic water bottles, food containers, and dental sealants. BPA levels in humans are measurable and significant (Ben-Jonathan and Steinmetz, 1998) and occur in ∼93% of the population (Group, 2013). BPA is effective at extremely low (nM) doses (Ben-Jonathan and Steinmetz, 1998) which correspond to the median value reported in US streams (Kolpin et al., 2002). BPA levels can be measured in human tissues and fluids (Chapin et al., 2008; Vandenberg et al., 2010; Lakind and Naiman, 2011) and, significantly, BPA is able to cross the placenta (Takahashi and Oishi, 2000; Chapin et al., 2008; Vandenberg et al., 2009) resulting in measurable fetal levels (Schonfelder et al., 2002; Chapin et al., 2008). There are no reports documenting developmental effects of BPA on humans (Chapin et al., 2008); however, *in utero* exposure (Chapin et al., 2008) causes a variety of behavioral deficits (Farabollini et al., 1999; Jasarevic et al., 2011; Kim et al., 2011; Wolstenholme et al., 2012) and is linked to childhood asthma (Nakajima et al., 2012) in rodents.

Our lab, and others, have reported the deleterious effects of exposure to BPA on the visual system. BPA targets neuroendocrine systems as a weak E2 agonist that binds and activates both ERs (Chung et al., 2011; Cano-Nicolau et al., 2016) and GPER (Thomas and Dong, 2006). BPA is extremely effective at low concentrations (Ben-Jonathan and Steinmetz, 1998), with exposure causing hyperactivity (Saili et al., 2012; Kinch et al., 2015; Weber et al., 2015), reduced midbrain size (Tse et al., 2013), and reduced outgrowth of motor neurons (Wang et al., 2013) in zebrafish. Because ER and aromatase regulation are estrogen-dependent, BPA causes dramatic overexpression of aromatase (Chung et al., 2011; Cano-Nicolau et al., 2016) and ER mRNA (Kishida et al., 2001), resulting in abnormally high estrogen signaling with likely adverse effects on nervous system development and function (Cano-Nicolau et al., 2016). For example, acute (24–48 h) BPA exposure in embryonic zebrafish causes defects in otolith formation (Gibert et al., 2011) and decreases in hair cell survival and regeneration (Hayashi et al., 2015), demonstrating that short-term exposure to BPA can have deleterious effects on sensory systems. Specific to the visual system, a chronic 120-day exposure in embryonic zebrafish (2 hpf) to BPS, a BPA analogue with similar estrogenic actions (Qiu et al., 2016), decreased tracking ability and the thickness of the ganglion cell layer and retina, and induced irregular arrangement of photoreceptor cells (Liu et al., 2017). Lastly, acute (24 h) exposure to BPA in larval zebrafish aged 72 hpf and 7 dpf resulted in changes in eye diameter and visually guided behaviors that were evident 1–2 weeks after removal from treatment (Crowley-Perry et al., 2021). These findings suggest that short- and long-term exposure to BPA can evoke both immediate and sustained effects on sensory systems, including the visual system.

Ethinyl-estradiol (EE2) is another estrogen receptor agonist that has been tested in zebrafish. EE2 is a synthetic derivative of endogenous E2 and, due to its wide use as a constituent in oral contraceptives, reaches aquatic environments through wastewater effluents (Vilela et al., 2021); agricultural and aquaculture runoff are other sources (Tang et al., 2021). EE2 concentration in surface waters varies, ranging up to 62 ng/L (Versonnen and Janssen, 2004), and it is consistently identified worldwide make it a serious environmental contaminant (Tang et al., 2021). EE2 exhibits higher potency and ER binding affinity than E2 (Aten and Eisenfeld, 1982; Denny et al., 2009), thus eliciting estrogenic effects at and below levels detected in the environment. There are no available epidemiological reports of EE2 and its effects on human sensory systems. However, EE2 binds to teleost and mammalian ERs (Aten and Eisenfeld, 1982), and environmentally relevant levels adversely affect fish (Nikoleris et al., 2016; Tang et al., 2021; Ramirez-Montero et al., 2022).

Embryonic zebrafish at 8–10 dpf exposed to picomolar concentrations of EE2 (10–1,000 p.m.) between 1 and 7 dpf of development have significantly inhibited axonal nerve and hair cell regeneration, suggesting direct impairments to nervous and sensory system development (Nasri et al., 2021). Similar 7-day EE2 exposures using pM to low nM concentrations caused significant overexpression of brain AroB and ERα/β transcripts in juvenile Atlantic salmon (Lyssimachou et al., 2006) and 7 dpf zebrafish (Cano-Nicolau et al., 2016; Nasri et al., 2021). Specific EE2 effects have also been observed in retina, where a 32-day exposure to low nanomolar concentrations (4–100 ng/L) decreased the outer and inner plexiform layers and total retinal thickness of minnows assessed at 28 days post-hatch (Alcaraz et al., 2021), an effect likely attributed to disrupted and/or heightened estrogen signaling.

Tributyltin (TBT) is an EDC that targets estrogenic pathways, but with opposite effects to BPA and EE2. TBT is an organotin compound used commonly as a biocide in antifouling paints applied to boats and marine structures and was historically found at high concentrations in aquatic environments (Mcallister and Kime, 2003). Though the International Maritime Organization banned the use of TBT in anti-fouling paints in 2008 (Showalter and Savarese, 2004; Gipperth, 2009; IMO, 2019), which lead to reduced environmental levels (Liang et al., 2017) and wildlife recovery (Jones and Ross, 2018), recent reports identify spikes in TBT levels (µg/g) in coastal areas off of Latin America, Norway, and Panama (Batista-Andrade et al., 2018; Schoyen et al., 2019; Castro et al., 2021) and TBT-based paint is still sold (Uc-Peraza et al., 2022), suggesting continued exposure. TBT is not readily biodegradable (Mcallister and Kime, 2003) and, once ingested, bioaccumulates and crosses the blood brain barrier, concentrating in areas of the brain that receive sensory inputs (Roulea et al., 2003).

TBT exposure in humans is not well studied, though exposure is thought to occur through consumption of contaminated fish or shellfish (Chien et al., 2002; Antizar-Ladislao, 2008). Such dietary intake of TBT has been measured worldwide and TBT is reported to inhibit placental aromatase (reviewed in Antizar-Ladislao, 2008), suggesting an impact on development.

TBT exposure causes a range of adverse effects, including increasing oxidative stress, triggering an immune response, reducing neurotransmitter synthesis/levels, increasing lipid accumulation, and altering liver function (Zhang CN. et al., 2017; Zhang J. et al., 2017; Ortiz-Villanueva et al., 2018; Barbosa et al., 2019; Li and Li, 2021; Shi et al., 2021). Relevant to this review is that TBT is also a known aromatase inhibitor that prevents the synthesis of E2 and decreases AroB expression in zebrafish brain (Lyssimachou et al., 2006). Plasma levels of testosterone are correspondingly increased, leading to deleterious effects on the reproductive system and population sex ratios. High levels of imposex in gastropod mollusks (Schoyen et al., 2019) and masculinization in fish (Mcallister and Kime, 2003; Santos et al., 2006; Mcginnis and Crivello, 2011) occur after TBT exposure. Within the visual system, embryonic fish (<8 hpf) transiently exposed to TBT exhibit abnormal eye growth (Hano et al., 2007) and apoptosis of retinal neurons (Dong et al., 2006). Degeneration and abnormal ordering of retinal layers has also been observed in larvae exposed to TBT for 10 days (Fent and Meier, 1992). Additionally, transient 24-h exposure to TBT during development alters visually guided optomotor responses (OMRs) measured 1-week after removal from treatment (Bernardo and Connaughton, 2022). Effects were age-dependent, with reduced OMRs occurring if TBT exposure occurred at 72 hpf or 7 dpf; reduced eye diameters were also observed when exposure occurred at 7 dpf.

Exposure studies using the pharmaceutical EDC 4-hydroxy-androstendione (4-OH-A or Formestane), another potent aromatase inhibitor, provide further support for the role of E2 in visual system development. A 3-day application of 4-OH-A to 48 hpf zebrafish prevented expression of normal sensory motor behaviors, including swimming movement, tactile response, fin movement, and eye movement (Nelson et al., 2008). Co-application with E2 at a concentration determined to be optimal for the transcriptional activation of ERs (Menuet et al., 2002) and upregulation of AroB mRNA (Kishida et al., 2001) rescued all sensory responses (Nelson et al., 2008), pointing to a key functional role of E2 signaling via ERs in sensory system development. Additionally, acute, 24-h exposure to 4-OH-A significantly decreased eye diameter in 7 dpf zebrafish (Gould et al., 2019). We also observed that the visual system effects of 4-OH-A persist into adulthood, as a 24-h exposure at 24 hpf, 72 hpf, and 7 dpf larval zebrafish resulted in significantly decreased visually guided optomotor responses in adults (3–4 months removed from treatment) (Gould et al., 2017), suggesting that even a brief disruption to estrogen signaling during development can have effects on maturation and long-term function. Taken together, these studies indicate that modulating E2 signaling via EDCs imposes both immediate and long-term effects on visual system development and function at a wide range of concentrations, developmental timepoints, and exposure durations.

### Role of Thyroid Hormones in Visual Function

Similar to estrogenic signaling, thyroid hormone signaling also coincides with retinal development (Figure 1). However, compared to E2, THs have a more direct involvement in retinal neurogenesis as they are required for neuronal maturation and cell fate of cone photoreceptors (Harpavat and Cepko, 2003; Roberts et al., 2006). In particular, TH binding to TRβ2 determines correct expression of cone opsins in both zebrafish (Harpavat and Cepko, 2003; Suzuki et al., 2013; Volkov et al., 2020) and rodents (Roberts et al., 2006). During development, zebrafish cones can express one of seven different opsins: lws1 (R1) or lws2 (R2) (red cones); rh2-1/rh2-2 (G1) or rh2-3 (G3) (green cones); sws2 (B1) or B2 (blue cones); sws1 (UV cones) (Takechi and Kawamura, 2005; Endeman et al., 2013; Nelson et al., 2019). *trβ2* expression is specifically required for expression of the red cone opsin (Suzuki et al., 2013) lws1 (Suzuki et al., 2013; Mackin et al., 2019; Volkov et al., 2020). Consequently, knockdown of *trβ2* reduces the number of red cones (Suzuki et al., 2013); zebrafish *thrb* mutants, as larvae or adults, have an anatomical loss of red cones (Volkov et al., 2020), which is associated with reduced response to red light and a loss of red cone inputs to the ERG (Deveau et al., 2020). Exposing zebrafish larvae to T3 from 2 to 4 dpf increased expression and distribution of lws1 (Mackin et al., 2019), consistent with effects in TRβ2 mutants. Interestingly, juveniles exposed to T4 from 26–31 dpf did not display a difference in lws1 expression (Mackin et al., 2019), though lws2 was altered. However, TH application upregulated *cyp27c1* in zebrafish juveniles (Mackin et al., 2019; Volkov et al., 2020). *cyp27c1* codes for the enzyme that converts vitamin A1 (the chromophore bound to opsin in zebrafish) to vitamin A2 in the retinal pigment epithelium (Allison et al., 2004; Enright et al., 2015; Volkov et al., 2020), consistent with a TH-induced shift toward longer wavelength sensitivity (Mackin et al., 2019; Volkov et al., 2020).

Though a major effect of *trβ2* expression is found in red cones, other cone types are also affected by changes in expression of this TR. For example, reductions in *trβ2* leads to an increase in the number of cones expressing UV opsin in zebrafish (Suzuki et al., 2013; Volkov et al., 2020), similar to the increase in S-cone number observed in TRβ2-null mice (Roberts et al., 2006), suggesting *trβ2*, and TH signaling, determines L-cone vs. UV-cone fate (Suzuki et al., 2013). Exposing *trβ2* mutant zebrafish larvae (2–4 dpf) to T3 caused a dose dependent increase in *rh2-2* and *rh2-3* expression (Mackin et al., 2019) in green cones, which was observed physiologically as reduced green sensitivity and a shift to a longer peak wavelength in photopic ERG recordings (Deveau et al., 2020), another example of a TH-induced shift to longer wavelength sensitivity. These results in zebrafish agree with those from human retinal organoid cultures, which show that TH binding to TRβ2 is required for L/M cone development (Eldred et al., 2018).

The thyroid axis is very sensitive to environmental chemicals. Many identified contaminants are able to affect this system and all levels are sensitive to disruption (Howdeshell, 2002; Boas et al., 2006; Patrick, 2009; Kohrle and Fradrich, 2021). Clinically, and experimentally, two EDCs are used to block TH synthesis: methimazole (MMI) or propylthiouracil (PTU). Both compounds are used as a treatment for hyperthyroidism (Yu et al., 2020) as they reduce the activity of thyroid peroxidase, the enzyme that catalyzes binding of iodine to tyrosine (Ohtaki et al., 1996); PTU also prevents formation of T4 from thyroglobulin (Bohnsack and Kahana, 2013). Song et al. (2017) performed a meta-analysis to assess the risk of congenital abnormalities in children born to mothers prescribed MMI vs. PTU during pregnancy. They conclude that MMI exposure resulted in a greater risk of congenital malformations, compared to mothers taking PTU (Song et al., 2017). Furter, disruption of or reduced TH signaling during pregnancy causes abnormal brain development and/or cognitive impairments (Patrick, 2009; Noyes et al., 2019). These deleterious effects can extend to “brain derivatives” that include retina, cochlea, and pacemaker cells (Howdeshell, 2002).

Exposing zebrafish embryos to 0.3 mM MMI between 60 and 72 hpf causes smaller eye diameters at 65 hpf which corresponded to a thinner GCL and IPL in treated retinas (Reider and Connaughton, 2014). Other neuronal, pharyngeal, and esophageal anomalies were also reported in zebrafish embryonically exposed to MMI (Komoike et al., 2013). These latter effects are similar to anomalies resulting from *in utero* exposure in humans (Komoike et al., 2013). MMI also reduces TH levels in adult rodents and reduces expression of Dio3 and Dio2 (Glaschke et al., 2011), suggesting not only reduced overall synthesis of TH, but a reduced ability to convert/activate circulating TH.

Exposure to PTU from 0 to 5 dpf reduced eye size in zebrafish larvae and alters optokinetic responses (Baumann et al., 2016; Gothie et al., 2019); effects correlated with PTU-induced downregulation of TR expression (Baumann et al., 2016). Subsequent microarray analysis revealed PTU exposure caused downregulation of phototransduction-related genes coding for opsins, phosphodiesterase, and arrestin (Baumann et al., 2019). In fact, of the genes involved in sensory perception, expression of >90% were found to be downregulated by PTU. The number of downregulated genes remained high when measured after a 3-day removal from treatment, though the levels of downregulation were less (Baumann et al., 2019), suggesting differential sensitivity to specific genes and long-term impacts of exposure.

TBBPA (tetrabromobisphenol-A) has also been used to examine TH signaling. TBBPA can bind to TR as either an agonist or antagonist, depending on the concentration used. TBBPA exposure reduced eye size and altered OKR in zebrafish larvae exposed from 0 to 5 dpf (Baumann et al., 2016; Gothie et al., 2019). The effect of TBBPA exposure on expression of specific genes was variable (Baumann et al., 2016), consistent with agonistic and/or antagonistic effects of this compound. Indeed, though opsin expression was upregulated after a 5-day exposure to TBBPA (from 0 to 5 dpf), overall TBBPA exposure caused more general effects than PTU (Baumann et al., 2019). However, compared to PTU-induced downregulation of genes involved in sensory perception, >80% were upregulated by TBBPA. Following 3 days of recovery/removal from treatment, opsin gene expression was still upregulated and detectable in TBBPA treated fish (Baumann et al., 2019).

### Summarizing E2 and TH Effects Identifies Crosstalk Between Estrogenic and Thyroidogenic Pathways

**FIGURE 2:**
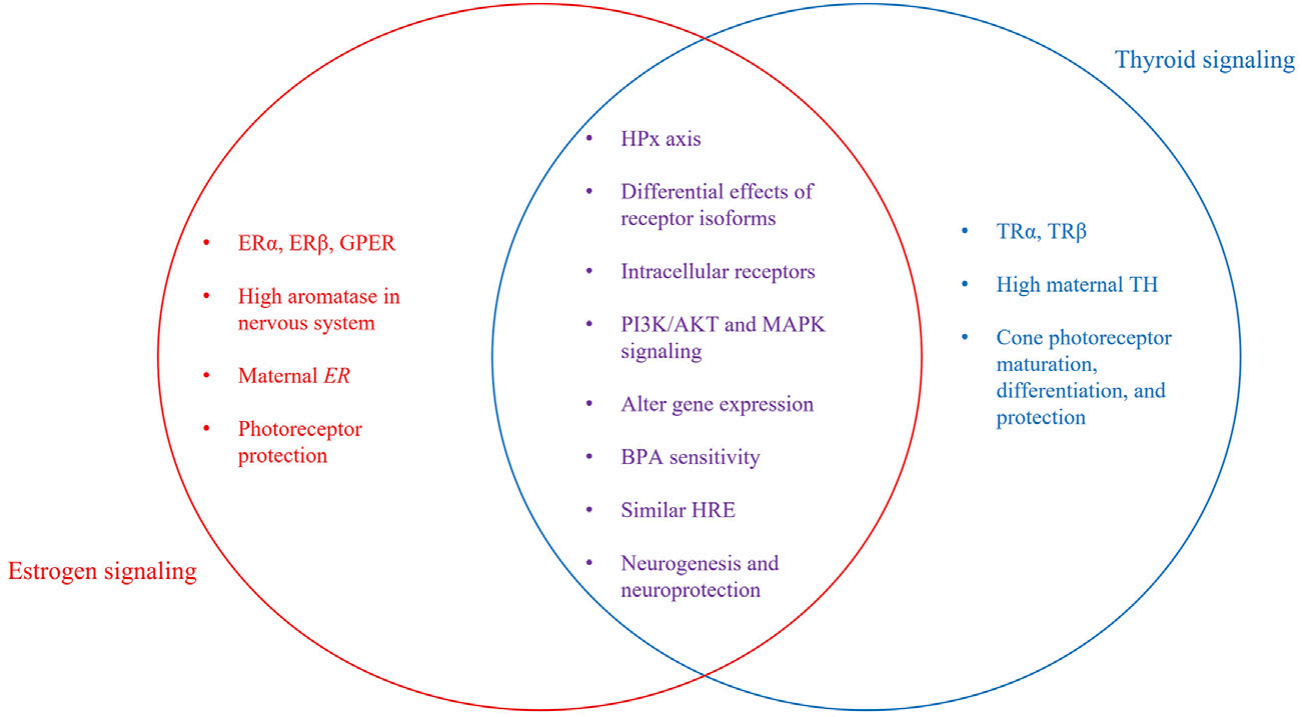
Independent and interactive pathway components. Venn Diagram showing individual and interactive aspects of estrogen and thyroid signaling. Shared characteristics between both signaling pathways include the involvement of the hypothalamus-pituitary axis, intracellular receptors, some intracellular signaling molecules, and effects on gene transcription. Both E2 and TH are required for neurogenesis, including development of the visual system. Photoreceptors are the retinal cell type most sensitive, as TH is required for correct development and organization of cones. E2 is neuroprotective, preventing cell loss due to light-damage or disease.

E2 and TH-based signaling pathways have many similarities (Figure 2). These similarities, though highlighted in zebrafish, are highly conserved across vertebrates. Both hormones are released from glands that receive stimulation through the hypothalamic-pituitary axis and both cross the plasma membrane of cells, bind intracellular receptors, initiate overlapping cytoplasmic signaling cascades, and interact with hormone response elements (HREs) to influence gene expression. Significantly, the half-sites of thyroid response elements (TREs) and estrogen response elements (EREs) exhibit striking sequence similarities in various promoters (Glass et al., 1988; Scott et al., 1997; Vasudevan et al., 2002). TRs have been shown to bind the consensus ERE with high affinity, preventing ERα-ERE interaction and consequently ERα-mediated transcription (Glass et al., 1988; Vasudevan et al., 2001; Vasudevan et al., 2002). There is evidence that ERs can also take part in this competition by binding TREs to suppress the effect of T3 on target promoters (Yarwood et al., 1993) and mediate strong estrogen-dependent activation of transcription (Graupner et al., 1991). Therefore, it appears that competition between ERs and TRs can lead to antagonizing effects.

One of the significant published reports related to cross talk between E2 and TH signaling relates to the shared sensitivity to BPA. As noted above, BPA is a weak estrogen agonist; however, it is also an antagonist of TR that prevents binding of T3 (Moriyama et al., 2002; Zoeller et al., 2005; Jung et al., 2007; Heimeier et al., 2009), thereby inhibiting negative feedback by TH and increasing serum T4 levels (Zoeller et al., 2005). BPA is a better antagonist for TRβ than TRα (Zoeller et al., 2005). Importantly, binding of BPA to TH receptors occurs at relatively high (>10 μM) BPA doses (Moriyama et al., 2002; Zoeller et al., 2005; Vandenberg et al., 2009). Low doses of BPA are reported to increase androgen receptor mRNA expression (Richter et al., 2007) but not to have anti-androgenic activity *in vivo* (Chapin et al., 2008). These interactions between TH and E2 signaling pathways, coupled to maternally derived *TH* and *ER* transcripts, their localization in the same tissues and the ability of those tissues to locally regulate hormonal actions, suggests TH and E2 signaling could mediate specific developmental events, such as retinal development.

Both hormones are present in the developing retina and synthesized locally within retinal tissue. In teleosts, the localization of neural aromatase, ERs, and GPER within embryonic and adult retina, and the functional deficits observed upon experimental estrogenic modulation, points to a key role of E2 in the visual system. Further, the importance of TRβ2 in cone photoreceptors and the strong early presence of maternal TH suggest a role for TH in the visual system. At 24 hpf, when the retina begins to develop, both *thraa* and *thrb* are expressed and TH levels are high due to maternally derived hormones in yolk. *AroB* and *ER* transcripts are detectable in retina at 24 hpf (Mouriec et al., 2009). Over the next ∼12 h, thyroglobulin (Alt et al., 2006) and gper (Mangiamele et al., 2017) expression is detected and the optic nerve leaves the eye (Steurmer et al., 1988). By 48–50 hpf, amacrine and horizontal cells in the INL appear, opsin expression begins, and *thrb* expression is seen in retina. When hatching occurs, retina and thyroid are fully functional and E2 signaling is functional.

Though thyroid and estrogen signaling have been examined for decades, there are still effects/mechanisms of these hormones that are poorly understood. Further their interaction(s) and influence(s) on each other is clearly complex and even less understood. However, considering the consequences of TH and E2 dysregulation, crosstalk in signaling, and developmental co-localization of receptors presented in this review, it is likely that these hormones work synergistically in the development, maturation, and function of the visual system. It is also likely that other sensory systems are impacted in a similar manner and future work should address these questions.
